# Posterior pelvic ring involvement detected with CT taken within a week of admission in acute fragility fractures of the pelvis (FFP) does not predict failure of conservative treatment: a retrospective cohort study

**DOI:** 10.1186/s12891-023-06439-1

**Published:** 2023-04-22

**Authors:** Guy Putzeys, Thomas Dekeyser, Patrick Garré, Tim Chesser, Hans Pottel

**Affiliations:** 1 Orthopedic and Trauma Department, AZ Groeninge hospital, Kortrijk, Belgium; 2 School of Medicine, UZ Leuven, Leuven, Belgium; 3 Department of data management, AZ Groeninge hospital, Kortrijk, Belgium; 4 North Bristol HHS Trust, Bristol, UK; 5 Department of Public Health and Primary Care, KULeuven KULAK, Kortrijk, Belgium

**Keywords:** Low energy pelvic fractures, Secondary surgery, Secondary displacement, Early CT, Elderly

## Abstract

**Background:**

Acute low energy pubic rami fractures in the elderly receive primarily conservative treatment. There is debate to what extent posterior ring involvement, which is detected superiorly by CT compared to X-ray, has an impact on outcome and may require modified treatment. We want to demonstrate if posterior ring involvement has an influence on different types of outcome in primarily conservatively treated acute FFP, questioning the usefulness of early CT. Additionally we analysed the early fracture pattern in cases where conservative treatment failed with need for secondary surgery.

**Methods:**

A retrospective cohort study of 155 consecutive patients, recruited between 2009 and 2016, aged over 65 years diagnosed with an acute LE-PFr on X-ray at the emergency department of a single, level-one trauma centre and receiving an early CT. A set of outcome parameters was compared between patients with an isolated pubic rami fracture (CTia) and patients who had a combined posterior pelvic ring fracture (CTcp).

**Results:**

There were 155 patients of whom 85.2% were female with a mean age of 83 years. 76.8% of patients living at home returned home and 15.5% moved to a nursing home. Mortality rate during hospitalisation was 6.4% and 14.8% at one year post-trauma. Secondary fracture displacement occurred in 22.6%. Secondary surgery was performed in 6 cases (3.9%). Median hospitalisation length of stay was 21 days (range 0 to 112 days). There was no significant association between the subgroups and change in residential status (p = 0.65), complications during hospitalisation (p = 0.75), mortality rate during admission (p = 0.75) and at 1 year (p = 0.88), readmission within 30 days (p = 0.46) and need for secondary surgery (p = 0.2). There was a significant increased median length of stay (p = 0.011) and rate of secondary displacement (p = 0.015) in subgroup CTcp. Secondary displacement had no impact on in-hospital complications (p = 0.7) nor mortality rate during admission (p = 0.79) or at 1 year (0.77). Early CT in patients who underwent secondary surgery showed stable B2.1 lesions in 4 of 6 cases.

**Conclusions:**

Our data suggest that early CT in patients with conservatively treated acute LE-PFr in order to detect posterior lesions, has limited value in predicting failure of conservative treatment.

**Supplementary Information:**

The online version contains supplementary material available at 10.1186/s12891-023-06439-1.

## Introduction

Low-energy or fragility fractures are fractures which result from mechanical forces that would not ordinarily result in a fracture. The World Health Organization (WHO) has quantified this as forces equivalent to a fall from a standing height or less [1]. Osteoporosis is a major factor for fragile bone and is predominantly seen in the elderly. With an increasing proportion of elderly in our Western population, the incidence of low energy fractures has been shown to increase [2–4] a trend also observed in pelvic fragility fractures [5–11].

Low energy pelvic fractures are known to have a significant clinical impact and may lead to a decrease in mobility, an increase in social dependency [12–14] and one-year mortality [6, 9, 15–19, 20].

FFP are in general classified as having an anterior and/or posterior component where the FFP with an isolated anterior component are described as stable and those with a posterior component have a decreased stability [21, 22].

In line with this anatomical instability concept in FFP, some clinical studies suggest that a FFP with a posterior component score worse for some or most outcome measurements [13, 18, 23] however this could not be reproduced by others showing no significant difference in outcome [17, 24–27].

Further differentiating between the different types of FFP with posterior injury Pol Rommens reported worse outcome in III and IV [28].

Low energy pelvic injuries in the elderly are usually diagnosed at the emergency department through a pelvic Xray showing pubic rami fractures. Standard pelvic radiographies however have a low sensitivity to detect posterior pelvic involvement. In contrast to X-ray slicing imaging techniques such as computed tomography (CT) and MRI have been shown to be highly sensitive to detect posterior involvement [29–35, 36] which led some authors to recommend the use of standard early CT when X-ray shows pubic rami fractures [7, 36–39].

The increased awareness of posterior lesion with the use of early CT and the significant clinical impact of conservatively treated FFP with a posterior component reported by some authors encouraged early surgical stabilisation in some centres, [37, 40, 41, 39]. Nevertheless Noser states that mortality and in-hospital complications remain high among patients with FFP even when receiving early surgery [42].

In our centre, we increased the use of early CT in FFP from 2009 onwards in order to detect posterior pelvic lesions however without changing our treatment strategy of primary conservative treatment. During the recruitment period, CT based fracture classification was within the hospital orthopaedic department thought to be clinically useful (increasing awareness for potential complications if an additional posterior lesion was present) however requesting CT at the emergency department was left to discretion of the emergency physician or geriatrician once the patient was admitted. At the start of this study patients were usually admitted at the orthopaedic ward, however from the third year of the study onwards due to hospital policy changes, patients were almost exclusively admitted to the geriatric department. As primary endpoint of our study we searched if a posterior pelvic injury detected with early CT in acute LE pubic rami fractures changed patient outcome. As secondary endpoints we analysed the impact of secondary displacement and severity of posterior involvement on outcome and the fracture appearance on primary imaging in cases which had secondary surgery. Our hypothesis is that detecting posterior pelvic injury with early CT has no significant impact on outcome and does not predict failure of conservative treatment. Our study was approved by the ethics board of AZ Groeninge Kortrijk (B396201730892).

## Materials and methods

This study is a retrospective cohort study with data prospectively collected in a consecutive way. It was conducted in a single, level one trauma centre, AZ Groeninge hospital in Kortrijk, Belgium. Data were retrieved from the German DGU Pelvic Injury Registry, containing data from all patients admitted to our hospital with pelvic and acetabular fractures between 2009 and 2016 [43]. Additional data were searched for in patient files, the hospital ICD 9 and 10 database, the hospital medical insurance database (data on surgery) and the Belgian national insurance database (data on mortality).

**Fig. 1:**
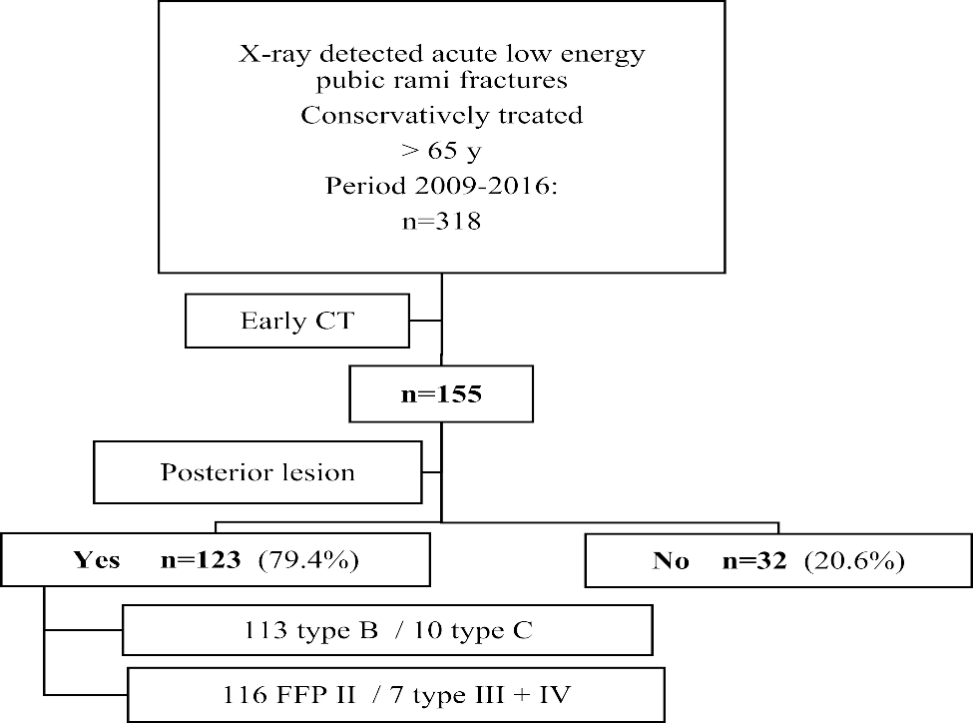
Inclusion criteria cohort and diagnostic flowchart

Inclusion criteria are (1) Admission at the emergency department (ED) with pubic rami fractures diagnosed on standard pelvic X-ray (2) Low energy trauma defined as falls from standing height or lower (e.g. falls from a chair, out of bed). (3) Acute trauma and (4) Elderly above 65 years. 5 Availability of early CT images of the pelvis (Fig. 1). We excluded atraumatic fractures, high energy traumas, pelvic ring fractures combined with acetabular or hip fractures, patients with negative, no or only X-rays, isolated posterior lesion, or patients who had primary surgery (two cases). X-ray examination at the emergency department consisted of an AP pelvis in combination with an X-ray of the hip on the painful side.

The policy of the hospital is to admit patients with LE pelvic injuries even if they came from a nursing home. 155 subjects were included. Acute trauma is defined as within 7 days of admission. Early CT scan is defined as within 1 week after admission. The decision to perform a CT scan was left at the discretion of the emergency physician or the treating physician once the patient was hospitalised. Conservative treatment was the primary treatment of choice and was similar for all patients independent of fracture characteristics being adequate analgesics, bedrest, mobilisation and weightbearing as tolerated. Primary outcome variables are residential status after hospital dismissal; readmission within 30 days; length of stay (hospitalisation excluding, stay at rehabilitation); mortality rate during hospitalisation and at one year after trauma; complications during hospitalisation, secondary displacement, acute posttraumatic pelvic bleeding and need for secondary fracture surgery. Secondary displacement is defined as the least displacement seen on sequential pelvic X-rays. Secondary outcome is early CT appearance of cases where conservative treatment failed. Complications during hospitalisation were divided into 10 different categories being anemia, decubitus, cardiological, gastro-enterological, orthopaedic, pneumological, psycho neurological, urological, infectious, and other complications. Pelvic fracture classification was performed according to the Tile classification [21] and FFP classification [22]. The Tile classification consists of 3 groups (A, B and C) and 9 subgroups. The fragility fracture of the pelvis (FFP) classification which was not adopted in the DGU Pelvic Injury Registry during the abovementioned registration period has been applied retrospectively. It incorporates bilaterality, anterior and/or posterior lesion in combination with displacement and consists of 4 groups (I to IV) and 11 subgroups reflecting increasing instability with an ascending numerical value [22]. FFP Type I is an isolated anterior injury with Type Ia unilateral and Type Ib bilateral. FP Type II is a non-displaced posterior injury. Type IIa: isolated, non-displaced sacral fracture. Type IIb sacral crush with anterior disruption and Type IIc a non-displaced sacral, iliosacral or ilium fracture with anterior disruption. FFP Type III is a displaced unilateral posterior injury. Type IIIa at the iliac, Type IIIb at the iliosacral and Type IIIc at the sacral level. FFP Type IV is a displaced bilateral posterior injury. Type IVa: bilateral iliac fracture or bilateral iliosacral disruption. Type IVb: bilateral sacral fracture, spinopelvic dissociation. Type IVc: combination of different dorsal instabilities. Classification of the pelvic fractures was performed by the senior author. Need for secondary surgery was searched for in the hospital medical insurance database until 3 year after sustaining the fracture. Secondary surgery was defined as (1) surgery indicated when conservative treatment fails and (2) performed after primary hospitalisation. Database configuration was done by an independent researcher. Statistical analysis was done by an independent statistician. No prior sample size calculation was done. The size is determined by the selected time-period. Similarity between the 2 groups was analysed for age, gender and admission status. Primary outcomes as residential status after dismissal; readmission within 30 days; length of stay, pelvic bleeding, mortality rate during hospitalisation and at one year after trauma and need for secondary surgery were available on the complete cohort whereas data on complications during hospitalisation and secondary displacement were only available for a part of the cohort. Concerning the secondary endpoints, we compared outcomes between subgroups with and without secondary displacement and between FFP II and FFP III/IV injuries. Within the subgroup of secondary surgery the location of pain at admission and the initial classification based on primary imaging was described as was the location of pain and classification at time of surgery. Descriptive statistics for quantitative variables is presented as mean (SD) or median (IQR) depending on normality of the data. Hypotheses tested were related to comparisons between the CTcp and CTia groups. An independent T-test was used to compare continuous variables between groups. Pearson chi-square test was used to compare categorical variables between groups (association). For 2 × 2 frequency tables, and the comparison of independent proportions, Fisher’s Exact test was used. McNemar’s test for dependent proportions was used to compare the residential status at admission and discharge in each group. Statistical analyses were performed with SPSS 28.0.1.1 (IBM SPSS Statistics Subscription).

## Results

155 patients were included in this cohort study. Except for 3 patients, all were admitted. There were 132 female (89%) and 23 male (11%) subjects. Mean age was 83 years (range 65–102 y), with a standard deviation of 6.7 years. 138 patients (87.2%) were living at home of which 57 subjects independently and 81 depending on others for their care; 17 subjects (12.8%) were living in a nursing home. 137 fractures were sustained at home, 16 outdoors and 2 fractures occurred in the hospital. 129 accident type were a fall from standing height; 6 subjects fell from their bed, 4 had a fall from lower stairs ,2 subjects fell from a chair, 1 fell into a hole, 2 fell from a ladder, 11 not known. The injuries were isolated to the pelvis in 119 cases. In 36 cases there were other non-pelvic fractures associated. Median hospitalisation length of stay, excluding stay in a rehabilitation department was 21 days (range 0 to 112 days). Based on the pelvic Xray taken at the emergency department the extent of displacement at the level of the pubic rami was between 1 and 5 mm in 127 cases; between 6 and 10 mm in 21 cases, between 11 and 20 mm in 8 cases and 25 mm in one case.

(Table 1). Only 3 bilateral pubic fractures were seen on standard X-rays and an additional 3 were seen on CT. According to AO/Tile classification there were 32 type A (20.4%) of which 31 type A2; 1 type A3; 113 type B (72.6%) of which 109 type B.2 and 4 type B.3 and 10 type C fracture (7%) of which 8 type C1; 1 type C2; 1 type C3. According to the FFP classification there were 32 FFP I (20.6%) of which 31 Ia; 1 Ib; 116 FFP II (74.8%) of which 94 IIb and 22 IIc; 5 FFP III (3.2%) of which 3 IIIa and 2 IIIc and 2 FFP IV (1.3%) both type IVb (Table 2).

**Table 1:** Patient characteristics cohort (n = 155)

Age	Mean age 83 y ( range 65–102 ), SD of 6.7 y.
Gender	Female 132 (85.2%) Male 23 (14.8%)
Residential status	Home 138 (89%) Independent 57 Dependent 81 Nursing home 17 (11%)
Location of accident	Home 137 (88.4%) Outdoors 16 (10.3%) In hospital 2 (1.3%)
Type of mechanism	Fall from standing height 129 Fall from bed 6 Fall from lower stairs 4 Fall from a chair 2 Fall into a hole 1 Fall from ladder 2 Other or not known 11
Isolated pelvic fracture Other non-pelvic fractures associated	119 36
Length of stay (LOS) *	Median = 21 days, IQR = 13.5–28.5 days

*length of stay is limited to stay in acute care hospital, stay at a rehabilitation department is not included

SD = standard deviation; IQR = interquartile range

**Table 2:** Fracture classification (n = 155 subjects)

AO/OTA^a^	n (%)	Subcategory	FFP ^b^	n (%)	Subcategory
A	32 (20.4%)	31 A2 / 1 A3	FFP I	32 (20.6%)	31 Ia / 1 Ib
B	113 (72.6%)	109 B2 / 4 B3	FFP II	116 (74.8%)	94 IIb / 22 IIc
C	10 ( 7%)	8 C1 /1C2 / 1 C3	FFP III	5 (3.2%)	3 IIIa / 2 IIIc
			FFP IV	2 (1.3%)	2 IVb
Total	155			155	

a: AO/OTA pelvic fracture classification : classification according to Tile/OTA (39)

b: FFP classification : classification of fragility fractures of the pelvis according to Rommens et al. (36)

There were 2 post traumatic anaemia due to pelvic fracture which needed treatment (1.3%): one in a patient with a FFP type IIc fracture who became hemodynamically unstable and was treated by coiling of the obturator artery, and one patient with a FFP type IIb fracture who was hemodynamically stable and was treated by transfusion a few days after admission. In total 92 complications (data available on 131 patients) were registered during hospitalisation of which 4 cardiological; 14 gastro-enterological; 7 orthopaedic; 10 pneumological; 7 psycho neurological; 15 urological complications; 10 infections; 5 anemia; 3 decubitus and 17 other complications (Table 3). Of the 138 patients living at home 106 subjects (76.8%) were dismissed to their home and 24 subjects (15.5%) were dismissed to a nursing home ( Table 4). Only 2 out of 155 patients were readmitted within 30 days after dismissal. Mortality during hospitalisation was 6.4% (n = 10). Cause of death: 6 respiratory, 2 cerebral, 1 cardial complication and 1 choking on food. One-year mortality rate was 14.8% (n = 23). 35 (22.6%) had secondary fracture displacement (Data available on 140 patients; 9.3% of the cohort). Median hospitalisation length of stay was 21 days (range 0 to 112 days; IQR = 13.5–28.5 days). There was no significant difference between the subgroups in age, gender and residential status pre-hospitalization (Table 5). There was no significant association between the subgroups and change in residential status (p = 0.65), complications during hospitalisation (p = 0.75), mortality rate during admission (p = 0.75) and at 1 year (p = 0.88), readmission within 30 days (p = 0.46) and need for secondary surgery (p = 0.2). There was a significant increased median length of stay (p = 0.011) and rate of secondary displacement (p = 0.015) in subgroup CTcp ( Table 6). No association was found between secondary displacement and in-hospital complications (p = 0.7) nor mortality rate during admission (p = 0.79) or at 1 year (p = 0.77) (Table 7). FFP II did not differ significantly from FFP III/IV injuries regarding LOS (p = 0.57), complications during hospitalisation (p = 0.55), mortality rate at 1 year (p = 0.28), and need for secondary surgery (p = 0.23). Mortality rate during admission was however significantly worse for FFP III/IV injuries (p = 0.003) (Table 8) 6 out of 155 subjects (3.9%) had secondary surgery during the follow up period of at least three years. Surgeries were performed 20 to 106 days after initial trauma. At admission 4 of them were sacral crush fractures, one case was a bilateral complete sacral ala fracture who refused primary surgery. 5 out of 6 fractures showed progress of instability of which 4 evolved from B2.1 to bilateral sacral fractures type C3.1 and one from B2.1 to a unilateral sacral fracture. 5 out of 6 patients underwent secondary surgery for a complaint not present at admission and a fracture not depicted on early CT. Details are shown in Table 9.

**Table 3:** Complications during admission *

	n	%
cardiological	4	3.7
gastro-enterological	14	12.9
orthopaedic	7	6.4
pneumological	10	9.2
psycho neurological	7	6.4
urological	15	13.8
infections	10	9.2
anemia	5	4,6
decubitus	3	2.7
other	17	15,6
total	92	100

* data on complications available on 131 patients

**Table 4:** Residential status at admission and discharge

Residential status	n = 155	RTORS*	Dismissal to nursing home	Deceased
Living at home	138	106 (76.8%)	24 (15.5%)	6 + 2 : 8 (5.2%)°
Nursing home	17	15 (88%)		2 (12%)

*RTORS: return to original residential status

°6 + 2 : 6 died during hospitalisation, 2 during rehabilitation

**Table 5:** Comparison of baseline characteristics between both subgroups

	2/Isolated anterior injury n = 32	1/Combined Posterior Injury n = 123	P value (chi-square test)
Age (mean)	84	82.9	0.201*
Gender (F/M)	25/7	107/16	0.209
Admission status (1/2/3/4)	15 / 14 / 0 / 3	42 / 67 /4 /10	0.429

* independent samples T-Test

1: at home independent 2: at home dependent 3: at nursing home independent 4: at nursing home independent

**Table 6:** Comparison of outcome between subgroups without and with combined posterior injury

	Isolated anterior injury n = 32	Combined Posterior Injury n = 123	P value (chi-square test)
Change in resid. status n = 155	5/32 (15.6%)	19/123 (15.4%)	0.65
LOS median [IQR] in days N = 155	16 (8.75-25)	22 (15–31)	0.011*
Complications during hospitalisation n = 92 (70.2%) on 131	7/22 = (63.6%)	31 /109 = (72.5%)	0.75
Mortality rate during admission n = 8 (5.2%) on 155	2/32 (6.25%)	6/123 (4.8%)	0.755
Mortality rate at 1 year n = 23 ( 13.7%) on 155	5/32 (15.6%)	18/123 (14.6%)	0.888
Secondary displacement n = 35 (25%) on 140	2/28 (7.1%)	33/112 (29.4%)	0.015
Readmission within 30 days n = 2 (1.2%) on 155	0/32 (0%)	2/123 (1.6%)	0.468
Need for sec surgery n = 6 (3.9%) on 155	0/32 (0%)	6/123 (4.9%)	0.203

* independent samples T-Test

**Table 7:** Comparison of outcome between subgroup without and with secondary displacement *

	No sec displacement	Sec displacement	P value (chi-square test)
Need for secondary surgery n = 6 on 140	2/105	4/35	0.016
Complications during hospitalisation n = 35 on 119	27/89	8/30	0.703
Mortality rate at 1 year n = 18 on 140	14/105	4/35	0.771
Mortality rate during admission n = 5 on 140	4/105	1/35	0.793

* data on sec displacement available on 140 patients

**Table 8:** Comparison of outcome between subgroup FFP II and subgroup FFP III/IV

	FFP II	FFP III/IV	P value (chi-square test)
LOS (mean)	24.37	27.86	0.571
Need for secondary surgery	5/116	1/7	0.234
Complications during hospitalisation	29/104	2/5	0.557
Mortality rate at 1 year	16/116	2/7	0.283
Mortality rate during admission	4/116	2/7	0.003
Secondary displacement	29/105	4/7	0.097
Readmission within 30 days	2/116	0/7	0.726

**Table 9:** Characteristics of patients who had secondary surgery

Gender	Age	Location pelvic pain on admission	Characteristics primary fracture ^a^	Timing Surgery (days)	Location pelvic pain & onset(weeks)^b^	Preop CT characteristics ^c^	Sec. displ. pubic rami (mm) ^d^
F	83	Posterior Right	Complete Right sacral ala Left pubic ramus C1.3 / IIc	106	Posterior Left 5 wks	Complete Bilateral sacral ala Bilateral pubic rami C3.3 / IVb	0
F	76	Posterior Left	Complete Bilateral sacral ala Left pubic ramus C3.3 / IVb	20	Posterior Left Continuous	Complete Bilateral sacral ala Left pubic ramus C3.3 / IVb	0
F	88	Anterior Right	Incomplete Right sacral ala Right pubic ramus B2.1 / IIb	106	Anterior Left 4 wks	Complete Bilateral sacral ala Bilateral pubic rami C3.3 / IVb	7
F	85	Posterior Right	Incomplete Right sacral ala Right pubic ramus B2.1 / IIb	64	Posterior Left 7 wks	Complete Bilateral sacral ala Right pubic rami C3.3 / IVb	15
F	88	Anterior Left	Incomplete Left sacral ala Left pubic ramus B2.1 / IIb	58	Anterior Right 4 wks	Complete Left sacral ala Bilateral pubic rami C1.3 / IIIc	4
F	88	Anterior Right	Incomplete Left sacral ala Right pubic ramus B2.1 / IIb	50	Posterior Right 5 wks	Complete Bilateral sacral ala Bilat eral pubic rami C3.3 / IVb	10

^a^ refers to the characteristics of the primary fracture : (first line) the completeness of the sacral ala fracture and its side (second line) the side of the pubic rami fracture and (third line) the resultant pelvic fracture classification

^b^ refers to : (first line) location of pain for which secondary surgery was performed and (second line) timing of onset of pain after the initial trauma in weeks.

^c^ refers to the reclassification of the fracture type prior to the secondary surgery : (first line) the sacral ala fracture and its side (second line) the pubic rami fracture side and (third line) the resultant pelvic fracture classification

^d^ secondary displacement of pubic rami fractures measured in millimetre as the difference between the primary XRay and the preoperative XRay

## Discussion

With this cohort study of consecutive acute LE pubic rami fractures in patients ≥ 65 year of age, we were able to show that posterior involvement detected with early CT has no significant impact on a number of short- and longterm outcome variables when treated conservatively.

Similar to our study, Natoli [24] evaluates the clinical utility of advanced imaging of the pelvis to identify posterior ring injuries. In his retrospective study on 87 patients above 60 years of age with primarily conservative treated low-energy FFP he compares an Xray only group of 45 patients with an advanced imaging group of 42 patients (10 MRIs, 32 CTs) of which 57% had a posterior injury and showed no clinical advantage of advanced imaging at 6- and 12 week follow-up. No patient underwent surgical intervention by 12-week follow-up and all patients with a minimum of 6-week follow-up were ambulatory. However the cohort consisted only of the more stable B2 fractures possibly favouring a good outcome and information was only available on middle term missing potential long term complications.

Several outcome studies on FFP have shown a decreased autonomy and increased mortality in comparison to uninjured patients [15, 16, 18, 44, 45] however within the group of FFP the impact of a posterior component show conflicting results.

Early studies with bone scintigraphy mentioning outcome in the presence of an additional posterior pelvic lesion conclude there is no impact on type of treatment [46, 47]. However only a small number of patients were included or they used less well documented outcome variables.

Other papers using CT as a diagnostic tool similarly report a limited impact [17, 24–27].

**Mears** [17] reporting on outcome of 181 conservatively treated Low energy fractures of the pelvic ring in patients older than 65 y, average age 85 y, of which 110 were hospitalized, compared different types of fractures and their displacement. They state that there was no significant association between fracture type and mortality at any point of time nor use of ambulatory aids, nor ability to remain in their own dwelling nor rate of complications during hospitalisation nor length of stay.

In contrast some studies suggest that FFP with a posterior component have a worse clinical outcome to a lesser or greater extent. Alnaib [23] in a prospective study of 54 conservatively treated low energy fractures of the pelvic ring, in patients older than 60 y, with average age of 87, report a mixed impact of fracture type (Combined pubic rami and sacral osteoporotic fractures) on outcome. He found no significant relationship in mobility and discharge destination, however a significantly higher rate of admission to hospital and longer length of stay in presence of a posterior fracture component. Loggers [13] In his retrospective study on 117 pubic rami fractures in patients older than 65 y, median age of 83, all primarily treated conservatively, reports that the presence of a posterior component did not significantly affect mortality at any point in time nor recovery to original mobility status, the independency status, a return to the original residency and pain at any point of time, but did significantly increase admission rate and complication rate during admission and a longer length of stay. However CT was only available in 34 patients (29% of the total cohort) of which 23 (67%), had a combined posterior lesion, making analysis less reliable.

Our median hospitalisation LOS was 21 days which is longer in comparison to most other reports on FFP. A possible explanation for this longer hospital stay is the admission policy of the hospital. From 2011 onwards patients with FFP were admitted at the geriatric ward. Papers reporting on patients with FFP admitted at geriatric wards report a substantially longer hospital stay compared to papers reporting on hospitalisation at an orthopaedic ward. Maier [16] reports a mean LOS of 15.2 days; Morris [19] 21.3 days and Taillandier [48] 45 ± 28 days versus Hill [12] who reports a mean LOS of 9.3 days, Mears [17] 5.9 days, Natoli [24] 4.25 days, W.A. van Dijk [44] 10 days and Osterhoff [49] 12 days or 8 days depending on the treatment type. Our reported longer LOS when a posterior component is present is in line with the findings of Alnaib [23] and Loggers [13].

We had a high incidence of secondary displacement of up to 25% in the complete cohort in comparison to other papers [24, 50]. Rommens [50] reported a FP of only 14.2% in the complete cohort, but when narrowing down to patients with an unfavourable clinical evolution ( 51 / 148 or 34.4% ) a second CT scan showed FP in nearly 40% (= 21/51 ).

More in line with our results in a recent study of Ueda [51], fracture progression was reported in as much as 30.4% of their cohort of 79 conservatively treated low energy FFP during their follow up until consolidation.

In our series secondary displacement was significantly increased in the presence of a posterior lesion (29.5% vs. 7.1%) which is expected given the presumed increased instability however it did not affect the rate of hospital complications nor rate of mortality during hospitalisation (p = 0.2887), in line with Natoli [24] who could not show dependence of a posterior ring injury within the subgroup of advanced imaging having any displacement > 1 cm at both the time of presentation and 6-weeks follow-up.

Secondary displacement did however predict a higher rate of secondary surgery suggesting the need for X-ray follow up in patients with FFP however we were not able to show a significant difference within the FFP group with posterior involvement between FFP II and FFPII/IV (p = 0.23).

Analysing the 6 FFP which underwent secondary surgery, 4 out of 6 were classified as stable IIb pelvic injuries at admission but they evolved over time to an unstable type III or IV FFP, a phenomena called by Rommens et al. “progress of instability or fracture progression” [52]. A possible explanation could be that early CT misses occult fractures in the acute setting. At the other hand only two out of 30 unstable pelvic injuries detected with early CT (22 x IIC, 5x type III and 2 x IV fractures) received secondary operative treatment. Interestingly those two cases did not show secondary displacement. These data correspond with the paper of **Rommens** [22] who states that operative treatment was performed mainly in delayed diagnosed III and IV fractures.

Similarly Höch [53] could not find influencing factors leading to failure of nonoperative treatment such as fracture pattern or classification in a cohort study of 128 patients with FFP aged ≥ 65 with lateral compression fractures of the Pelvis type B2.1 (90%) and B3.3 (10%) where he compares nonoperative with operative treatment.

The incidence of secondary surgery in low energy pubic rami fracture is rarely reported and is highly variable. Loggers [13] in his retrospective study on 117 pubic rami fractures in patients older than 65 y, all primarily treated conservatively, reports a similar incidence of 3% of secondary surgeries after dismissal however lacking detailed information on fracture type, only that they all had a posterior component. In contrast Höch [53] reports a high incidence of 18% of secondary surgery (14 cases) within the nonoperative subgroup of 77 patients. In his paper in 2013 [22] and his review paper in 2017 [54] Rommens recommends surgical treatment even in FFP II, mostly diagnosed in an acute setting, in order to avoid a more problematic revalidation with increased risk for instability when conservatively treated.

Our data however question this more recent trend towards early primary surgery in FFP with a posterior component reflected in several papers [22, 40, 41, 52].

Although several studies show that surgery in FFP can be safe and efficient [55, 56–58] there is still a debate about indication and timing of surgery.

More recent papers comparing operative with nonoperative treatment are less conclusive. [42, 49, 57, a 59, 28].

Wilson [60] states in his systematic PRISMA review of operative management of fragility fractures of the pelvis (17 eligible studies with 766 patients) that the quality of evidence was poor with no good quality randomised trials. The limited availability of non-operative comparators made it difficult to draw firm conclusions about the efficacy of surgical over non-surgical management.

Besides the option of surgical treatment of FFP modification of conservative treatment has been shown to improve outcome in the treatment of FFP [61, 62]. In a cohort study of 132 elderly patients with conservatively treated FFP, Kanakaris [60] showed that introducing a specific clinical algorithm in combination with antiosteoporotic drugs resulted in less malunions and non-unions and a higher chance for return to pre-injury mobility state.

In our cohort there were 2 patients with pelvic haemorrhage which required treatment (1.3%), one of whom needed urgent coiling of a bleeding obturator artery, the other was treated with a transfusion. Life-threatening bleeding from FFP are very rare [63, 64]. There is a lack of evidence-based recommendations concerning the optimal screening and management of the bleeding in elderly patients with FFP however monitoring these patients regardless of whether displaced or non-displaced FFP is recommended especially when under anticoagulation [63, 64].

The limitations of this study are inherent with its retrospective study design reporting associations and not causations. One can question the specificity of the used parameters to represent the clinical picture in comparison to “mobility” “quality of life” or “pain level” which we were not able to trace in a reliable way. We recognize that residential status at discharge and mortality do not only depend on FFP but are also dependent on comorbidities, social environment, availability of home care or rehabilitation units. The clinical follow up was limited to the primary hospitalisation period but we were able to detect the incidence of secondary surgery up to three years after the initial trauma. Strengths of our study are the more defined inclusion criteria with focus on acute FFP representing the most frequent clinical entity of FFP seen at the emergency department. Except for 3 all patients were admitted to our hospital allowing detailed description of their medical and social situation. Data were meticulously registered in an extended version of the German DGU Pelvic Injury Registry with pelvic fracture specific data.

## Conclusion

Our data suggest that posterior pelvic ring involvement detected with early CT in acute fragility fractures of the pelvis (FFP) does not predict failure of conservative treatment. One can question the usefulness of early CT scans from the pelvis during the initial workup after acute low energy pelvic trauma in the elderly minimizing cost and radiation exposure.

## Electronic supplementary material

Below is the link to the electronic supplementary material.

Supplementary Material 1

## Data Availability

All data generated or analysed during this study are available in a supplementary information Excel file.
